# Mind Matters: A Critical Look at Malaysia's Postnatal Depression Policy for Women's Mental Health

**DOI:** 10.1089/whr.2023.0027

**Published:** 2023-07-28

**Authors:** Aishah Siddiqah Alimuddin, Nuur Asyikin Mohd Shukor, Shean Yih Soh, Rosnadia Suainbon, Asma Assa'edah Mahmud, Farah Deena Abdul Samad, Nurazah Ismail, Muna Hamiza Asiff

**Affiliations:** ^1^ Department of Psychiatry, Faculty of Medicine and Health Sciences, Universiti Putra Malaysia, Serdang, Malaysia.; ^2^ Department of Psychiatry, Hospital Canselor Tuanku Muhriz UKM, Jalan Yaacob Latiff, Bandar Tun Razak, Cheras, Malaysia.; ^3^ Psychiatry Unit, Faculty of Medicine and Defence Health, National Defence University of Malaysia, Kem Sungai Besi, Malaysia.; ^4^ Psychiatry Unit, Department of Medicine, Faculty of Medicine and Health Sciences, Universiti Sains Islam Malaysia, Nilai, Malaysia.; ^5^ Psychological Medicine Unit, Faculty of Medicine, Universiti Sultan Zainal Abidin (UniSZA), Jalan Sultan Mahmud, Kuala Terengganu, Malaysia.

**Keywords:** postnatal depression, policy, women's mental health, prevention, intervention

## Abstract

**Introduction:**

This policy brief examines the national health and action plans, laws and regulations, public health policies, and clinical practice guidelines in Malaysia on postnatal depression (PND).

**Methods:**

We examined and included 13 documents for the presence or lack of a statement of intent and/or actions related to caring for women at risk for or experiencing PND.

**Results:**

Although PND is actively researched and included in the clinical practice guidelines, no other policy documents mention PND.

**Conclusion:**

General recommendations to address this matter include channeling resources into developing care for PND, increasing advocacy work to reduce stigma, setting up appropriate training pathways for health care providers, and creating more roles and user-friendly modules for local volunteers to deliver mental health interventions.

## Introduction

Malaysia is a multicultural, multiethnic, and multireligious country with numerous nationalities. This beautiful country is home to a total of 32.7 million population, with 15.9 million being of the female gender.^1^ In the past decade in Malaysia, there has been a steady growth of work on preventing and treating mental health conditions and mental illness.^2^ As the country moves toward equality in gender in all aspects, health issues focusing on women are increasingly highlighted. This includes the more specific issue surrounding postnatal depression (PND).

The prevalence of PND in Malaysia ranges from 14.3% to 31.7%,^3,4^ with increased cases due to increased awareness. Despite this, some members of the population remain ignorant or often have prejudices toward individuals with PND, calling them names such as “Meroyan” and considering them dangerous.^5^ A peek into the local dictionary (Dewan Bahasa Pustaka) reveals “Meroyan” as a state of “emotional instability after giving birth due to hormonal changes, causing the mother to be depressed or to act aggressively.^6^”

As a result, the term has a local colloquial connotation of implying a crazy woman who may put her child at risk, resulting in increased stigma, which causes a delay in help seeking for those suffering from postpartum depression and psychosis. These “labels” exemplify how much more work is required to break through to different pockets of the population.

According to *DSM 5*, PND is characterized by low mood, loss of interest, poor sleep and concentration, lethargy, loss of appetite and weight, guilt feelings, and/or thoughts of death, lasting for 2 weeks or more.^7^ This is further expanded in several PND guidelines to periods of up to 1 year and involves thoughts of inadequacy as a mother and infanticidal ideas.^8,9^

The extent of impairment for untreated PND is massive, as it involves the mother, the newborn, and other family members.^9,10^ At a higher level, PND also impacts health economics, increasing the loss of workdays for women affected and increasing expenditure on mental health treatment.^11,12^ Therefore, much attention is required to address the issue at a larger scale once and for all.

The World Health Organization (WHO), in collaboration with the United Nations Population Fund (UNFPA), has been calling for the use of policy to integrate maternal mental health care into services offered, especially in lower- and middle-income countries (LAMICs).^13^ One way to deal with this is by looking into the country's current policies, laws, regulations, and guidelines. The aim is to recognize the gaps in the existing documents and formulate a plan to improve them to improve this vital subject, which has not been adequately discussed in the past.

We examined the documents for a statement of intent and/or actions related to the care of women at risk for or experiencing PND based on groups on national health and action plans, laws and regulations, public health policies, and clinical practice guidelines from the year 2000 up to year 2022. We searched the electronic databases using the following keywords: postnatal depression, postpartum depression, maternal mental health, post-natal depression, post-partum depression, women, and mothers. When documents were not available electronically, we consulted with field experts from the judicial and health systems to gain insight into the records available within their archives. After a content analysis to interpret the data in the policy texts, we found 13 articles that we included in this policy brief.

## Policy Options and Implications

Malaysia's unique legal system reflects its cultural diversity and colonial history, with one of the few countries applying dual law.^14^ Islam is recognized as its official religion in Malaysia, but other religions can be practiced in harmony. Federal law is enacted and applied throughout the country, whereas state and Islamic laws are promulgated by the state and used within the particular state. Should any inconsistency exist between federal and state law, federal law precedes state law.^15^ A search on the laws and regulations in Malaysia applicable to mental health—specifically on PND—was fruitless.

Despite using multiple search keywords, no relevant law documents was found. We enquired about this with a group of law practitioners who agreed with our online search findings. However, they highlighted the presence of the Medical Act 1971 (Act 50) for general health provisions,^16^ and the Mental Health Act 2001 and Mental Health Regulation 2010, which encompasses the general provisions of managing a person with a mental disorder in Malaysia.^17,18^ However, these documents lack information on actions to prevent, detect, or treat PND. There are also no law provisions on services for women with PND.

Other acts concerning women in Malaysia include the Women and Girls Protection Act 1973 [which was repealed by Child Act 2001 (Act 611)], the Married Women Act 1957, and the Domestic Violence Act 1994 (Revised 2012).^19–21^ However, despite its widely known connection to intimate partner violence, they do not mention any specific provision on women's mental health, especially PND. Searching for maternal mental health laws in other countries quickly reveals that several states in the United States have Maternal Mental Health Acts or are coming up with one (*e.g*., New Moms Act 2022 in the 117th Congress Bill).^22^

These acts, however, do not translate to the developing countries in this region. The lack of laws and regulations on maternal mental health leads to delayed care delivery to this critical population group due to a lack of task forces and operational policies, human resources, and financial aid. This results in mortality and morbidity, which could easily be preventable with early identification and treatment, had there been a proper system in place.

In Malaysia, gender gap differences are measured using Malaysia Gender Gap Index (MGGI). MGGI analyzes the differences between men and women in four indices: economic participation and opportunity, educational attainment, health and survival, and political empowerment. A 1.0 (100%) score signifies that gender equality has been attained.^23^ In 2006, the gender gap was reduced to 0.1057,^24^ educational attainment to 0.0441, economic participation to 0.2455, and political empowerment to 0.5754.

Therefore, the national women's action development in 2009 focused on minimizing the gender gap further, in line with the national mission (2006–2020) of practicing equality in human capital for both men and women. Its target includes helping women address issues in economics, law, education, media, environment, sports, religion and culture, science and technology, and politics. It also highlights the importance of protecting women's rights and welfare, with women's well-being as the main agenda.

Numerous strategies were proposed concerning health promotion, women's friendly health services, improving maternal and child health services facilities, and developing a national women's health database. However, there was no statement of intent or emphasis given to mental health in the perinatal period or mental health in women, despite it being a high-stakes issue for women. Even in the 10th and 11th Malaysia Plans,^25,26^ despite the focus on empowering the population with noncommunicable diseases and plans on improving the quality of health care further, there was no discussion on women's mental health.

Understanding Malaysia's health sector will help us better understand the public health policy—Malaysia's health care system consists of public and private health sectors. Both sectors consist of primary care clinics and secondary/tertiary hospitals. Maternal health care in public health is divided into antenatal, perinatal, and postnatal care. Antenatal care is carried out mainly by the public health clinic (primary care setting). During the antenatal or perinatal period, complicated cases are referred to a secondary/tertiary hospital with specialist care for further assessment.

Deliveries are carried out in either secondary or tertiary hospitals. The postnatal care would be returned to the primary care setting. However, perinatal and postnatal care focused mainly on physical health. No relevant documents related to PND in Malaysia was found when a general policy search from various websites was done. Midwifery nurses carried out the delivery of postnatal care with postbasic training, which included the subject of PND.^27^ Owing to the nature of their work during the postnatal period, midwifery nurses are the best professionals to perform PND screening.

They work closely with mothers postdelivery due to the frequent home visits within the delivery month. However, studies have shown that these groups of nurses reported having inadequate PND knowledge and limited abilities to identify mothers with symptoms of PND, resulting in many not performing PND assessments and undiagnosed cases.^27^

Furthermore, when nurses are not involved directly in PND treatment, they have limited knowledge concerning the use of antidepressants in breastfeeding mothers and other options such as electroconvulsive therapy.^28^ It affects their capacity to provide counseling for mothers regarding treatment options. What is observable is that there is a theory–practice gap that exists where the nurses' knowledge is not reflected in their delivery of care. The main challenge that mental health professionals face is the lack of policy and engagement with obstetric professionals regarding issues of PND, resulting in the women diagnosed with PND being treated within the same psychiatric setting as other general patients.^28^

This poses a significant barrier to help-seeking behavior and further impacts the bonding with the newborn child because of the limitation of a public psychiatric setting that separates the mother and the infant. Ideally, what is best is to be able to provide treatment for the mother in an obstetric environment with a liaison psychiatry team working together to address the PND, which is clearly lacking, as it is not even considered part of the psychiatry operating policy.^2,29^

In Malaysia, the guideline for managing PND was discussed as part of the depression in the perinatal period in the 2nd edition national clinical practice guideline for managing major depressive disorder.^30^ This guideline recommended that screening for PND should be done between 6 and 12 weeks postpartum and repeated at least once within the first year postnatally with brief screening tools, such as Patient Health Questionnaire-2 (PHQ-2) or Whooley Questions. The same guideline also recommended using psychotherapy and other psychosocial intervention as the treatment for mild-to-moderate disease.

In contrast, pharmacotherapy should be used if the patient has a severe disorder. A safety profile of the use of various antidepressants during breastfeeding was included in the guide. Meanwhile, the Institute for Public Health of the Ministry of Health, Malaysia, also published its 2nd edition of the simple cognitive behavioral therapy module for treating PND named TIARA-MURNI.^31^ This module targeted to provide the outline of therapy using cognitive-behavioral therapy principles in treating mothers with postnatal mothers.

It laid down the rules of referral in the module, the rules, and the goals of the therapy sessions. The module consists of six sessions of therapy that are 30 minutes each, to be conducted once every week. The general schedule of the therapy session includes a 5-minute introduction or revision of the last session, a 10-minute learning and discussion, a 10-minute practical session, and the last 5 minutes of take-home messages and assignments. Each session in this therapy focuses on different skills and topics to be delivered and learned by the participants. They include:

- Focus on psychoeducation and introduction of the biopsychosocial model, the signs and symptoms of postnatal blues, PND, and postnatal psychosis, and the risk factors and treatment of PND. The homework is for participants to identify physical, cognitive, emotional, and behavioral changes during PND.
- Focus on relaxation techniques such as deep breathing exercises and mindfulness.
- Focus on identifying cognitive distortion and techniques to challenge negative thoughts.
- Focus on the relationship with the spouse, identification of issues within the family, and effective communication skills will be taught.
- Focus on the relationship with the baby by learning various interaction methods with the baby and exploring the emotion of being a mother.
- Focus on relapse prevention and summarize the whole module.

This module includes a copy of the Malay version of edinburgh postnatal depression scale and automatic thoughts questionnaires. The TIARA-MURNI module is a unique effort by the Ministry of Health Malaysia, allowing simple CBT to be learned and delivered by primary care providers without relying on clinical psychologist services, which may not be available to the large population of perinatal mothers. Accessibility to psychotherapy and cost-effectiveness of service are improved through this initiative.

Malaysia clinical practice guideline for major depressive disorder provides a basic and brief outline of PND management. However, it did not discuss how integrated care among various health care providers, such as obstetricians, psychiatrists, and primary care physicians, can be delivered and catered to the need of postnatal mothers. Furthermore, assessment of the mother's capacity to nurse the child, mother–infant interaction, infant's safety, and mother's risk are still lacking in the current CPG.

This is in contrast with the CPG for mood disorder published by Royal Australian and New Zealand College of Psychiatrists (RANZCP) and perinatal mental health care guideline by the Centre of Perinatal Excellence (COPE) of Australia,^32,33^ where these issues were outlined. Future editions of the Malaysia CPG may address these gaps or a separate guideline focusing on perinatal mental health may be beneficial to outline integrated care recommendations involving all the stakeholders.

## Actionable Recommendations

We acknowledge the challenges faced in formulating and implementing policies in Malaysia due to the limited resource allocation, stigma, and lack of professional expertise. However, an effective maternal mental health policy can help overcome these issues, allowing stakeholders to take the necessary steps to tackle them. Some general recommendations include channeling more resources into developing mental health care, specifically for PND, increasing advocacy work to reduce stigma, setting up appropriate training pathways for health care providers, and creating more roles and user-friendly modules for local volunteers to deliver mental health interventions.

More specific recommendations for this include:

(1) Innovating human resources making health care more accessible to the community
  (a) There are some programs that can be converted to modules that can be conducted by either artificial intelligence or transforming it into materials that can be done independently online. By doing so, patients who may have severe form of illness can have access to help allowing the service to focus on more severe case getting help sooner.
  (b) At the moment, the service provider is focused at the tertiary level. Task shifting practices should be a priority, whereby staff who are at primary care or the first health care provider to come in contact with patients is equipped with adequate skills and training to manage less severe cases. They are thought to identify potentially worrying cases that may need more help. Subsequently establishing a good line of communication between different centers, ensuring that consultation can be done smoothly.
  (c) Telemedicine consultations are implemented in cases where urgent consultations are needed but patients may be remote and have limited access to health care services, this can reduce the need for in-person visits.
  (d) Collaboration with nonprofit organization, other government agencies, and community stakeholders is vital to ensure that attempt to gather resources and coordinating work and manpower to ensure that the delivery of health is at an optimum and effective level.
(2) Assessment—Concerns have been raised about providing screening for all women in the perinatal period due to a lack of staffing to handle the possible overflow of cases found during screening. However, the first step in recognizing the gravity of the issue is by screening, and once the rising prevalence is noted, more allocations could be made to improve the service.
(3) Treatment—Developing a mother–baby unit caring exclusively for mother and child during a postnatal mental health crisis is deemed of utmost necessity to ensure better reception and delivery of a holistic treatment plan for patients with PND and their babies.
(4) Impact on other sectors—There may be unintended consequences for employment and child care sectors if this issue is not addressed, and, therefore, further discussion with the relevant stakeholders to help implement policies and guidelines holistically. Suggestion includes allowing extended maternity and paternity leaves and having child care centers within the workplace compound with shorter working hours for new parents while they adapt to parenthood.
(5) Policy level definitions and goals—Having a clear set of operational definitions for frequently used terms such as “disability,” “recovery,” “early interventions,” and “preventions” will help in understanding the requirements of each of these aspects and, therefore, create more robust goals to assist in achieving them.
(6) Systemic issues—Allocating financial resources for mental health services, including subsidized services in a government setting, and hastened insurance clearance for private services.
(7) Encouraging a stand-alone women's mental health policy and budget—Although it can be covered in education, social welfare, and general health policies, a specific policy for women's mental health is still needed to ensure much more robust work in the area.
(8) Engagement and involvement of stakeholders in psychoeducation and antistigma programs—Psychoeducation involves individual or group programs with motivational, educational, and behavioral techniques focused on knowledge and understanding of the disease symptoms, treatment, prognosis, and rehabilitation. It can be used as part of stigma-reduction strategies. Coinvolvement of the patient, caregivers, family members, and mental health care professionals is crucial for this program's success.
(9) Collaborative work within the multidisciplinary teams (*e.g*., psychiatry, obstetric and gynecology, and social welfare) in terms of drawing up guidelines for PND
(10) Reinforce the role of research and researchers in the policy process—creating methodologically sound and culturally compatible research on prevalence, treatment, and strategy evaluation to increase the visibility of women's mental health issues and provide real-time evidence-based solutions that could be adopted in policy and services planning.
(11) Regular evaluation of the available services and guidelines in the form of an audit to ensure they are still relevant and applicable to current times.

## Conclusion

Although PND is mentioned in a few operational-level policy articles, it is not covered in the national-level policies in Malaysia. This discrepancy will only cause a further gap in the prospect of health care improvement, especially concerning women's empowerment of their well-being. A coordinated effort requiring the collaboration of multisectors is necessary to ensure that half of the country's population continue to grow together in health and all other aspects of human rights to ensure we move forward to the ideal developed country.

## Abbreviations Used

COPE: Centre of Perinatal Excellence
LAMICs: lower- and middle-income countries
MGGI: Malaysia Gender Gap Index
PHQ-2: Patient Health Questionnaire-2
PND: postnatal depression
RANZCP: Royal Australian and New Zealand College of Psychiatrists
UNFPA: United Nations Population Fund
WHO: World Health Organization
